# Efficient shortcuts to adiabatic passage for three-dimensional entanglement generation via transitionless quantum driving

**DOI:** 10.1038/srep30929

**Published:** 2016-08-08

**Authors:** Shuang He, Shi-Lei Su, Dong-Yang Wang, Wen-Mei Sun, Cheng-Hua Bai, Ai-Dong Zhu, Hong-Fu Wang, Shou Zhang

**Affiliations:** 1 Department of Physics, College of Science, Yanbian University, Yanji, Jilin 133002, China; 2 School of Physical Science & Engineering and Key Laboratory of Materials Physics of Ministry of Education of China, Zhengzhou University, Zhengzhou 450052 China

## Abstract

We propose an effective scheme of shortcuts to adiabaticity for generating a three-dimensional entanglement of two atoms trapped in a cavity using the transitionless quantum driving (TQD) approach. The key point of this approach is to construct an effective Hamiltonian that drives the dynamics of a system along instantaneous eigenstates of a reference Hamiltonian to reproduce the same final state as that of an adiabatic process within a much shorter time. In this paper, the shortcuts to adiabatic passage are constructed by introducing two auxiliary excited levels in each atom and applying extra cavity modes and classical fields to drive the relevant transitions. Thereby, the three-dimensional entanglement is obtained with a faster rate than that in the adiabatic passage. Moreover, the influences of atomic spontaneous emission and photon loss on the fidelity are discussed by numerical simulation. The results show that the speed of entanglement implementation is greatly improved by the use of adiabatic shortcuts and that this entanglement implementation is robust against decoherence. This will be beneficial to the preparation of high-dimensional entanglement in experiment and provides the necessary conditions for the application of high-dimensional entangled states in quantum information processing.

Quantum entanglement is an essential resource for quantum computation and quantum communication that has many promising practical applications in quantum information processing (QIP). Two-dimensional entanglement is the most general quantum entanglement that is involved in many QIP tasks^1^,^2^,^3^,^4^,^5^,^6^,^7^,^8^,^9^,^10^,^11^,^12^ such as quantum computing^6^,^7^,^8^, teleportation^5^, cryptography^7^,^9^, and precision measurements^10^. However, the high-dimensional entanglement has many fundamental and practical advantages compared to its two-dimensional counterparts^13^. It not only demonstrates the violations of local realism but can also be used to enhance the security of quantum cryptography. Motivated by this, many schemes have been proposed to generate a high-dimensional entanglement^14^,^15^,^16^,^17^,^18^,^19^,^20^,^21^. For example, Shao *et al.* and Su *et al.* proposed schemes to create the three-dimensional entanglement by utilizing the dissipations of the physical system as the auxiliary resources^14^,^15^,^16^ and included the design of the non-resonant system in their schemes leading to a long evolution time. Li and Huang suggested a deterministic scheme to generate a three-dimensional entangled state in a resonant system via quantum Zeno dynamics, in which the time required to produce entanglement is very short compared to that required in dispersive protocols^17^. Wu *et al.* proposed a scheme to achieve the multi-particle three-dimensional entanglement state via adiabatic passage^18^. Although the adiabatic passage can effectively resist the fluctuations of the parameters, it requires a long time of dynamic evolution. Liang *et al.* proposed a scheme that combines the adiabatic passage with quantum Zeno dynamics to realize the three-dimensional entanglement^19^. Although the scheme is simplified by using Zeno dynamics, it inevitably leads to a long evolution time due to the adiabatic passage. To date, two experimental schemes have been proposed for generating a high-dimensional entanglement that takes advantage of the spatial modes of the electromagnetic field carrying orbital angular momentum^20^,^21^.

It is well known that the robustness of adiabatic passage against parameter fluctuations makes it a good choice for the realization of QIP. However, the required long evolution time is the key ingredient that makes it effective. In practice, however, a long evolution time may be a drawback that makes the method ineffective because the dissipation caused by decoherence, noise, and losses on the target state can increase with an increasing interaction time. Therefore, much attention has been devoted to improving the speed of the adiabatic passage, and the shortcuts to adiabaticity that arise in this situation. Several theoretical and experimental schemes have been proposed to realize the shortcuts to adiabaticity^22^,^23^,^24^,^25^,^26^,^27^,^28^,^29^,^30^,^31^,^32^,^33^,^34^. Two methods can be used to construct the shortcuts: the first is the inverse engineering based on the Lewis-Riesenfeld invariant (LR)^35^,^36^,^37^, and the second is the TQD proposed by Berry^38^,^39^,^40^,^41^. These two methods are strongly interrelated and are even potentially equivalent. The characteristic of the LR-based method is that the original Hamiltonian is not destroyed in the construction of the shortcuts, but in some cases, the fixed form of the invariants may be a weakness for the construction of the shortcuts. The TQD method provides an effective way to construct the counter-diabatic driving (CDD) Hamiltonian that accurately drives the instantaneous eigenstates of the original Hamiltonian. However, it was found that in practice, the designed CDD Hamiltonian is difficult to implement directly^42^,^43^,^44^,^45^,^46^. Several schemes have been proposed to overcome this obstacle; for instance, Chen *et al.* proposed a scheme to generate the Greenberger-Horne-Zeilinger (GHZ) state using quantum Zeno dynamics and TQD^28^. In 2016, Song *et al.* presented an interesting approach for the implementation of the physically feasible three-level TQD with multiple Schrödinger dynamics^47^. Inspired by the above works, in this study, we construct shortcuts to the adiabaticity of three-dimensional entanglement by introducing auxiliary levels and a large detuning condition to improve the generation efficiency and expand the application of three-dimensional entanglement in cavity quantum electrodynamics. Unlike ref. 28, we generate the three-dimensional entanglement state merely by applying the TQD method. This scheme can effectively speed up the generation of three-dimensional entanglement in the adiabatic passage. Moreover, our numerical simulation shows that the present scheme can reach a high fidelity under dissipation and can therefore be helpful in dealing with the tasks of fast quantum communication and computation.

## Results

### Basic model

**Figure 1:**
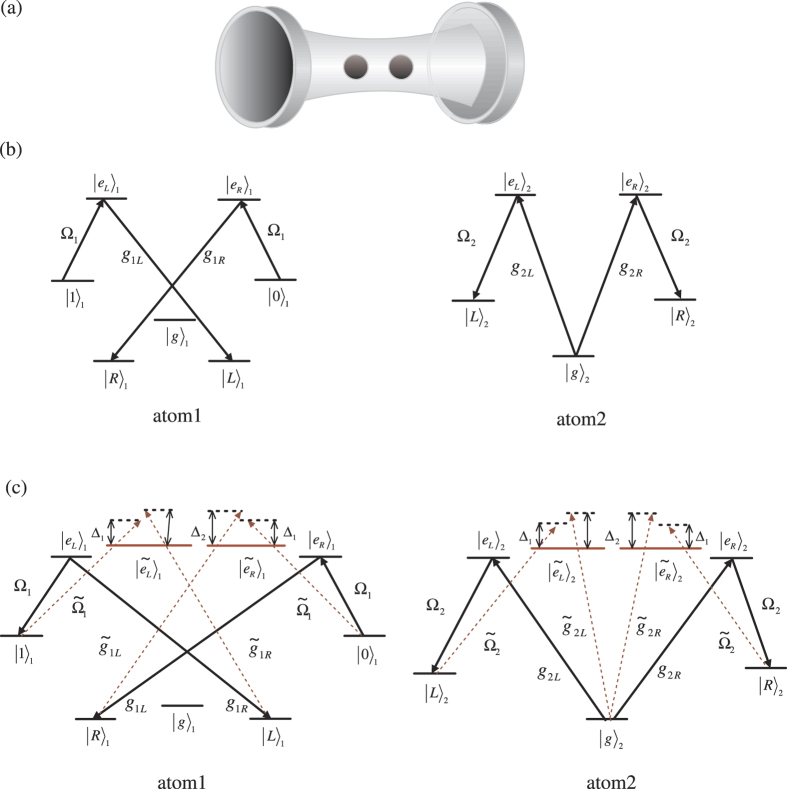
(**a**) Schematic view of the setup and two atoms are trapped in the cavity. (**b**) Level configuration of atom 1(2) for the original Hamiltonian. (**c**) Level configuration of atom 1(2) for the shortcuts to adiabatic passage.

We consider a multimode cavity in which two atoms are trapped as shown in Fig. 1(a). The atomic level configuration depicted in Fig. 1(b) was used by Wu *et al.*^18^. Atom 1 has two excited states |*e*_*j*_〉_1_ ( *j* = *L*, *R*, the same below) and five ground states |1〉_1_, |*R*〉_1_, |*g*〉_1_, |*L*〉_1_, and |0〉_1_, while atom 2 is a five-level system with three ground states |*R*〉_2_, |*g*〉_2_, |*L*〉_2_ and two excited states |*e*_*j*_〉_2_. For atom 1, the transitions |*e*_*L*_〉_1_ ↔ |1〉_1_ and |*e*_*R*_〉_1_ ↔ |0〉_1_ are driven by the classical fields with the same Rabi frequency Ω_1_(*t*), and the transition |*e*_*j*_〉_1_ ↔ | *j*〉_1_ is resonantly driven by the corresponding cavity mode *a*_1*j*_ and the coupling strength *g*_1*j*_. For atom 2, the transitions |*e*_*j*_〉_2_ ↔ |* j*〉_2_ are driven by the classical fields with the same Rabi frequency Ω_2_(*t*), and the transition |*e*_*j*_〉_2_ ↔ |*g*〉_2_ is resonantly driven by the corresponding cavity mode *a*_2*j*_ with the coupling strength *g*_2*j*_. The configuration described here can be obtained from the hyperfine structure of cold alkali-metal atoms^48^,^49^,^50^. Here we use two ^87^Rb atoms that have been cooled and trapped in a small optical cavity. For atom 1, 5^2^*S*_1/2_ ground level |*F* = 1, *m* = 2〉 (|*F* = 1, *m* = −2〉) can be used as the state |*L*〉 (|*R*〉) and |*F* = 2, *m* = 1〉 (|*F* = 2, *m* = −1〉) as |1〉 (|0〉), respectively. The 5^2^*P*_3/2_ excited level |*F*′ = 1, *m* = 1〉 (|*F*′ = 1, *m* = −1〉) can be used as the state |*e*_*L*_〉 (|*e*_*R*_〉). Other hyperfine levels in the ground-state manifold can be used as |*g*〉 for atom 1. For atom 2, the 5^2^*S*_1/2_ ground level |*F* = 1, *m* = 0〉, |*F* = 2, *m* = 2〉, |*F* = 2, *m* = −2〉 can be used as states |*g*〉, |*R*〉, and |*L*〉, respectively. The excited level |*F*′ = 1, *m* = 1〉 (|*F*′ = 1, *m* = −1〉) corresponds to |*e*_*L*_〉 (|*e*_*R*_〉). The total Hamiltonian in the interaction picture can be written as (*ħ* = 1)


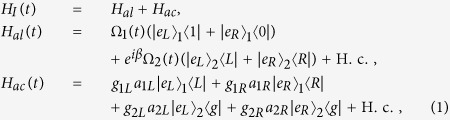


where *β* is the phase difference between the two time-dependent classical fields and we have assumed *β* = 3*π*/2 here; *a*_*ij*_ (*i* = 1, 2; *j* = *R*, *L*) is the annihilation operator for the corresponding cavity modes with *R*(*L*)-circular polarization, and *g*_*ij*_ (*i* = 1, 2; *j* = *R*, *L*) is the coupling strength between the corresponding cavity mode and the atom.

We now describe an idea for constructing the shortcuts to adiabatic passage to generate the three-dimensional entanglement between the two atoms; this can be written as


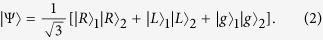


Initially, atom 1 is prepared in the state 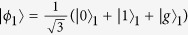, and atom 2 in state |*g*〉_2_, with both cavity modes in the vacuum state. To clearly illustrate the physical method for the shortcut, we first use the state |0〉_1_ |*g*〉_2_ |0〉_*c*_ as the example. In this situation, the system is restricted to the subspace spanned by


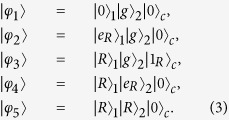


For simplicity, we assume *g*_*ij*_ = *g* (*i* = 1, 2; *j* = *R*, *L*) and assume the condition of weak-driving fields


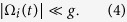


Then, the eigenstates |*ψ*_*n*_(*t*)〉 at the instantaneous time *t* and the corresponding eigenvalues *ξ*_*n*_(*t*) of *H*_*I*_(*t*) that obey the equation *H*_*I*_(*t*)|*ψ*_*n*_(*t*)〉 = *ξ*_*n*_(*t*)|*ψ*_*n*_(*t*)〉 can be derived analytically as


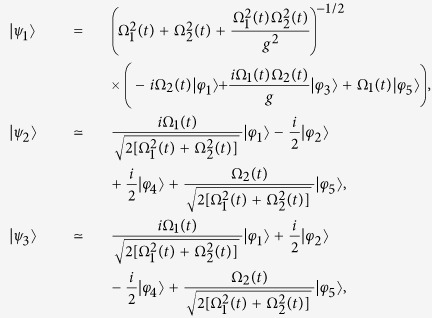



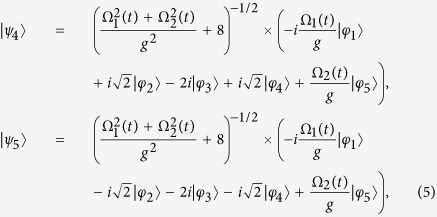


and


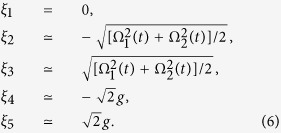


It can be seen that the eigenstate |*ψ*_1_(*t*)〉 is a dark state in the subspace with an eigenvalue of *ξ*_1_ = 0. If the adiabatic condition 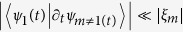 is fulfilled^51^, the initial state will undergo an evolution determined by |*ψ*_1_(*t*)〉, which neglects the probability of populating the |*φ*_3_〉 state during the entire evolution. Undoubtedly, the adiabatic passage is an effective method for implementing the transformation from the initial state to the final state, but it requires a long time to complete the evolution. This is undesirable due to decoherence. The shortcut to the adiabatic passage is a good choice for the acceleration of the adiabatic evolution in a nonadiabatic manner. The evolutions of the other two initial states will be interpreted later.

### Shortcuts for a generating three-dimensional entanglement of two atoms

The instantaneous eigenstates |*ψ*_*n*_(*t*)〉 for the Hamiltonian *H*_*I*_(*t*) do not satisfy the Schrodinger equation *i*∂_*t*_|*ψ*_*n*_(*t*)〉 = *H*_*I*_(*t*)|*ψ*_*n*_(*t*)〉. According to Berry’s general transitionless tracking algorithm, one can reverse engineer a Hamiltonian related to the original Hamiltonian *H*_*I*_(*t*), but drives the eigenstates exactly^40^. The Hamiltonian can be obtained by using 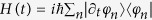 with |*φ*_*n*_〉 the eigenstates of original Hamiltonian *H*_*I*_(*t*); see the method section in detail. Substituting Eq. (5) into the above formula, we obtain the simplest Hamiltonian *H*(*t*) in the form


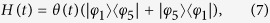


where


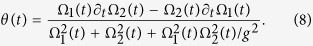


However, because the two atoms with double Λ level configurations in the original system are resonant with the cavity modes as well as with the classical lasers (see Fig. 1(a)), the two excited states of each atom are occupied with a considerable proportion of the population. It is difficult to realize the intended transitions between the ground states within the atoms. Thus, in practice, the direct implementation of the CDD Hamiltonian *H*(*t*) is still challenging, especially in multi-particle systems. It is necessary for us to construct an alternative physically feasible (APF) Hamiltonian equivalent to *H*(*t*).

To construct the APF Hamiltonian, two auxiliary levels must be introduced in each of the atoms described above, as depicted in Fig. 1(c). For atom 1, the 5^2^*P*_3/2_ excited levels |*F*′ = 2, ±2〉 of atom ^87^Rb can be used as the two auxiliary excited levels 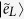 and 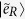, respectively. For atom 2, the excited levels 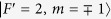 of 5^2^*P*_1/2_ can be used as the auxiliary levels 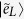 and 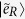, respectively. Correspondingly, two additional classical driving fields with Rabi frequencies 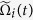 (*i* = 1, 2) and two auxiliary cavity field modes are introduced to drive the relevant transitions. The transition 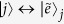 (* j* = *L*, *R*) of atom 1 and 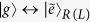 of atom 2 are coupled, respectively, to the auxiliary cavity modes with the coupling constant 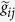 (*i* = 1, 2 and *j* = *R*, *L*) and detuning Δ_2_. The two classical laser fields are applied to drive the transition 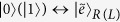 and 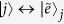 of atoms 1 and 2, respectively, with the same detuning Δ_1_. Under the rotating wave approximation, the auxiliary interaction Hamiltonian is (*ħ* = 1)


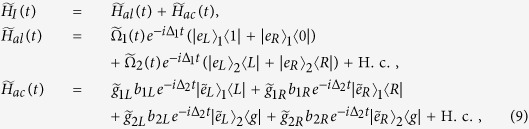


where *b*_*ij*_ (*i* = 1, 2; *j* = *R*, *L*) denotes the annihilation operator of the auxiliary cavity mode and the symbol H.c. means Hermitian conjugate. For simplicity, we have assumed 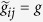 and 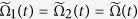. If the system is initially in the state |*φ*〉_1_, under the condition of a large detuning regime Δ_1_, 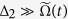, *g*, the level 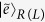 and the auxiliary cavity modes *b*_*R*(*L*)_ are virtually excited. Thus, we can adiabatically eliminate the excited states of the atoms and obtain the auxiliary effective Hamiltonian^52^,^53^,^54^,


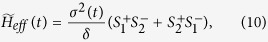


where 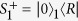, 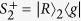, and 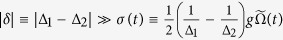. The effective Hamiltonian (10) is equivalent to the CDD Hamiltonian *H*(*t*) in Eq. (7) with


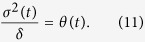


Hence, the Rabi frequency of the auxiliary laser field contributes to the construction of the APF Hamiltonian and can be determined from the original frequencies Ω_1_(*t*) and Ω_2_(*t*) as


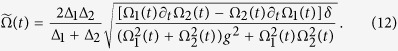


**Figure 2:**
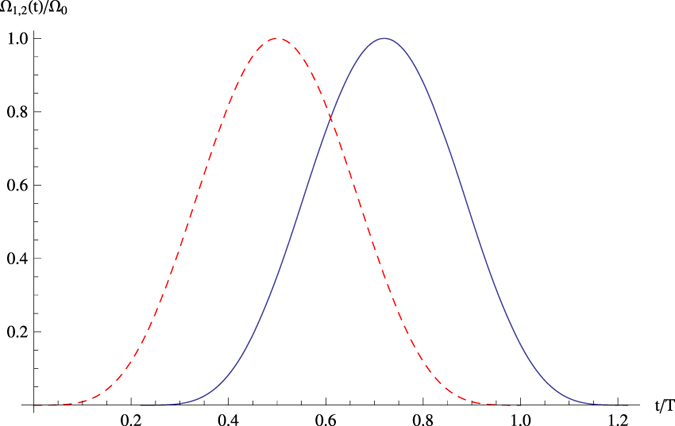
The shapes of Ω_1_(*t*)/Ω_0_ (solid blue line) and Ω_2_(*t*)/Ω_0_ (dashed red line) dependent on *t*/*T*, where Ω_1_(*t*) and Ω_2_(*t*) are defined by Eqs (22) and (23) with *τ* = 0.22 *T*.

To satisfy the adiabatic condition


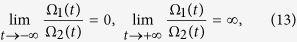


the Rabi frequencies Ω_1_(*t*) and Ω_2_(*t*) in the original Hamiltonian *H*_*I*_(*t*) can be chosen as


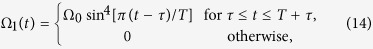


and


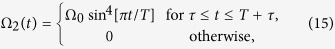


where Ω_0_ is the pulse amplitude, *τ* is the time delay, and *T* is the operating duration. Figure 2 shows Ω_1_(*t*)/Ω_0_ and Ω_2_(*t*)/Ω_0_ plotted as a function of *t*/*T* for a fixed value of time delay chosen for the best adiabatic passage. Applying this shortcut to the adiabatic passage, the initial state |0〉_1_ |*g*〉_2_ |0〉_*c*_ finally evolves to state |*R*〉_1_ |*R*〉_2_ |0〉_*c*_.

In contrast, if the initial state is |1〉_1_ |*g*〉_2_ |0〉_*c*_, the system is restricted to the subspace spanned by the basis vectors


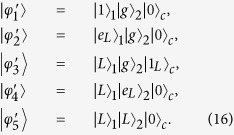


In this case, using the method described above, we can easily obtain the effective Hamiltonian


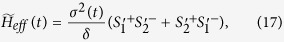


where 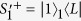, 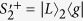. Finally, the system will evolve to the state |*L*〉_1_ |*L*〉_2_ |0〉_*c*_. Meanwhile, the initial state |*g*〉_1_ |*g*〉_2_ |0〉_*c*_ remains unchanged during the evolution due to the absence of excitation.

Considering all of the above cases, we can see that the two atoms in the initial state


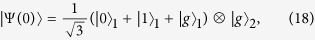


will evolve into the three-dimensional entangled state in Eq. (2), assisted by a vacuum cavity exploiting the shortcuts to adiabatic passage. All of the cavity modes finally stay in the vacuum states.

## Discussion

**Figure 3:**
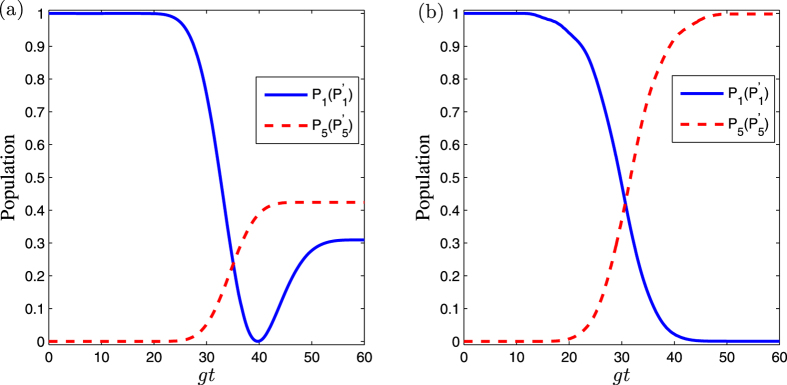
Time evolution of the population for the states 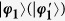 and 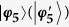 with Ω_0_ = 0.2 *g*, *T* = 50/*g*, and *τ* = 0.22 *T*. (**a**) governed by the original Hamiltonian *H*_*I*_(*t*), (**b**) governed by the APF Hamiltonian 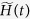 with Δ_1_ = 6 *g*, Δ_2_ = 7 *g*.

To prove the efficiency of the shortcuts assisted by the Hamiltonian 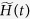, we use a contrast between the performance of the population transfer from the initial state to the final state driven by the APF Hamiltonian 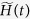 and that governed by the original Hamiltonian *H*_*I*_(*t*), as shown in Fig. 3.

The time-dependent population for any state |*φ*〉 is given by the relationship *P* = 〈*φ*|*ρ*(*t*)|*φ*〉, where *ρ*(*t*) is the corresponding time-dependent density operator. A comparison of Fig. 3(a,b) shows that the APF Hamiltonian 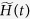 governs the system to achieve a near-perfect population transfer in a short interaction time, whereas the original Hamiltonian *H*_*I*_(*t*) does not show such an effect. This can be understood in physics. By introducing the auxiliary levels in each atom and driving the transitions between the auxiliary levels and the ground states with the cavity modes and classical fields, the interaction energy within the system is increased. This enhances the effective transition strength (or coupling strength) between the ground states (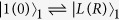 and 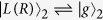), thereby greatly increasing the population probabilities of the target states and accelerating the entire process.

**Figure 4:**
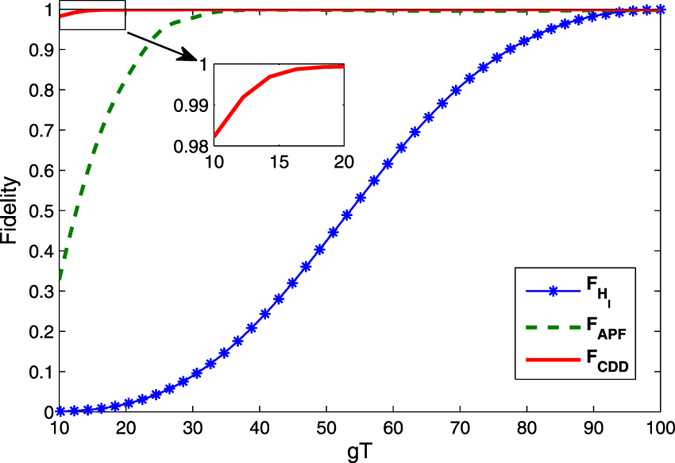
Fidelities of the three-dimensional entanglement state are shown as a function of *gT*. They are governed by CDD Hamiltonian *H*(*t*), APF Hamiltonian 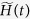 with Δ_1_ = 6 *g* and Δ_2_ = 7 *g* and original Hamiltonian *H*_*I*_(*t*), respectively. The Rabi frequencies Ω_1_(*t*) and Ω_2_(*t*) are defined by Eqs (22) and (23) with Ω_0_ = 0.2 *g*, *τ* = 0.22 *T*.

We can also compare the fidelities of the entangled states governed by the original Hamiltonian *H*_*I*_(*t*), *H*_*I*_(*t*) assisted by the APF Hamiltonian 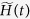 and those governed by the CDD Hamiltonian *H*(*t*). As shown in Fig. 4, as a fast and feasible experimental method, the fidelity for our shortcuts scheme can achieve the same perfect degree as that driven by the CDD Hamiltonian *F*_*CDD*_, with only a slightly longer time. Meanwhile, this process is much faster than the adiabatic passage.

**Figure 5:**
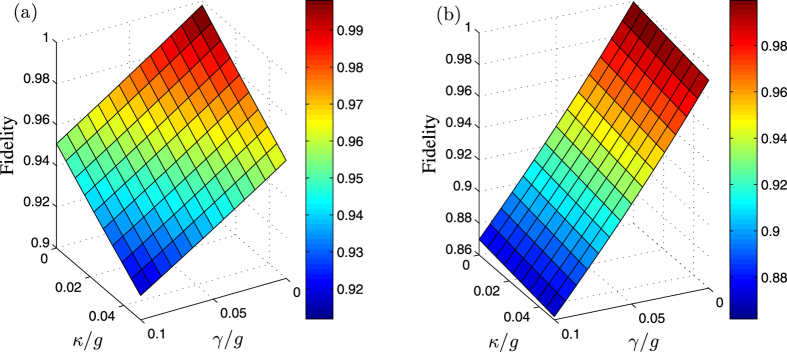
Fidelity of the target state as a function of *κ*/*g* and *γ*/*g*. The Rabi frequencies Ω_1_(*t*) and Ω_2_(*t*) are defined by Eqs (22) and (23) with Ω_0_ = 0.2 *g* and *τ* = 0.22 *T*. (**a**) governed by the APF Hamiltonian 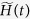 with Δ_1_ = 6 *g*, Δ_2_ = 7 *g*, *T* = 50/*g*. (**b**) governed by the original Hamiltonian *H*_*I*_(*t*) with *T* = 100/*g*.

In a realistic implementation, in addition to the operating speed requirements, the robustness of the scheme against the possible decoherence caused by atomic spontaneous emission *γ* and cavity decay *κ* should also be considered. Using the Lindblad master equation, we can simulate the fidelity of this scheme defined by 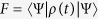 with the *ρ*(*t*) being the reduced density matrix of the final state. An examination of Fig. 5(a) shows that under the dissipative conditions, the intended entanglement state can be obtained with a high fidelity of more than 90% in the present shortcut scheme. Moreover, the fidelity increases with decreasing *γ* and *κ*, e.g., a fidelity 98.18% can be reached with *γ*/*g* = 0.014 and *κ*/*g* = 0.01. To reveal the effectiveness of the shortcuts, the fidelity of original scheme under the dissipation is shown in Fig. 5(b). Comparison of Fig. 5(a,b) shows that the original fidelity is always lower than that in the present shortcut to adiabatic passage under the same degree of dissipation factors (cavity decay and spontaneous emission). The spontaneous emission in the shortcuts to the adiabatic passage has a smaller influence than does that in the original scheme. Therefore, our present scheme is more robust.

The realistic problem related to the experiment is how to capture the two ^87^Rb atoms into the same cavity and control them precisely by using different laser pulses in the same cavity. The optical dipole trap (ODT) is one optimal candidate system for QIP using laser cooling techniques^55^,^56^,^57^,^58^,^59^,^60^. A quantum register composed of 5 qubits and the controllable Rabi oscillation for 5 qubits has been realized by using monochromatic microwave field to coherently control these atoms^61^. Kim and Saffman *et al.* constructed five one-dimensional linear ODTs with a spatial distance on the micron level between each other using diffractive optical elements and successfully realized 5 single-atom qubits with no mutual disturbing between any two qubits^62^. We can also use the technology of an atom conveyor belt^63^,^64^ to implement our present scheme. Chapman’s group developed this technique further with dual atom conveyor belts^65^,^66^ so that two single atoms confined in two optical lattices can be transferred to the designated positions in the cavity. These two lattices are sufficiently far apart along the direction perpendicular to the axis of the cavity that the probe beams excite atoms in only one of the lattices. The atoms are loaded simultaneously from the magneto-optical trap, but each lattice has independent translational control. Using strong focusing laser fields and detuning the frequencies, the required transitions can be realized.

## Conclusion

We have constructed a shortcut to the adiabatic passage for the three-dimensional entanglement of two atoms by using the TQD method. We construct a supplemental interaction Hamiltonian that is equivalent to the counter-diabatic Hamiltonian under a large detuning regime. The numerical simulation demonstrates that the scheme is fast and robust against the decoherence caused by atomic spontaneous emission and cavity decay. We have also discussed the feasibility of the scheme in experiment. In view of the high security of high-dimensional entanglement in quantum communication and quantum cryptography, our present scheme is expected to have practical applications in quantum communication.

## Methods

### Transitionless quantum driving

The starting point of TQD is an arbitrary time-dependent Hamiltonian *H*_0_(*t*) with the instantaneous eigenstates |*φ*_*n*_(*t*)〉 and energies *λ*_*n*_(*t*), given by


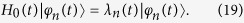


Under the adiabatic approximation, the state driven by *H*_0_(*t*) would be^36^


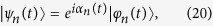


where the adiabatic phase including dynamical and geometric parts is


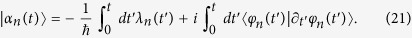


To find the Hamiltonian *H*(*t*) that drives the eigenstates |*φ*_*n*_(*t*)〉, we first define a time-dependent unitary operator


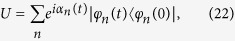


which obeys


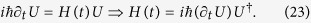


Then the Hamiltonian *H*(*t*) is obtained as


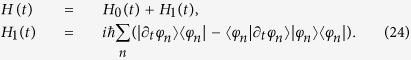


The simplest choice is *α*_*n*_ = 0, for which the bare states |*φ*_*n*_(*t*)〉 with no phase factors are driven by


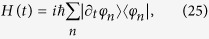


reflecting


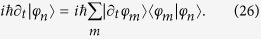


### Modelling of decoherence effects

The short evolution time is the striking characteristic of our scheme, but the evolution will inevitably suffer from decoherence. Therefore, we pay attention to the effects of decoherence on our entanglement generation. The main dissipation channels include the spontaneous emission of atoms and cavity decay. Considering all of these factors, the evolution of our scheme is governed by the following master equation


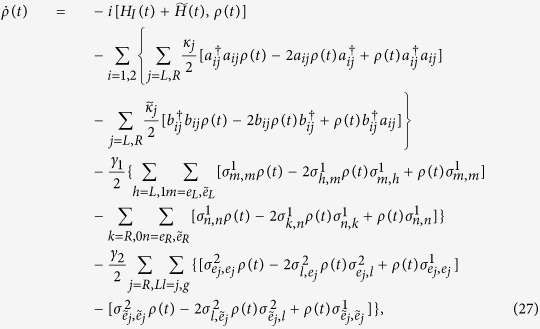


where *κ*_*R*(*L*)_ denotes the decay rates of cavity mode *R*(*L*), *γ*_1(2)_ denotes the spontaneous emission rate of atom 1(2) from 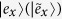 (*x* = *R*, *L*) to |*R*〉, |*L*〉, |*g*〉, respectively; 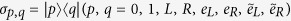 are the usual Pauli matrices. The fidelity of the three-dimensional entanglement state versus the ratios *γ*/*g* and *κ*/*g* has been shown in Fig. 5 where we have assumed 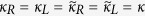, *γ*_1_ = *γ*/5, *γ*_2_ = *γ*/3 for simplicity. In experiments, the cavity QED parameters *g*/2*π* ≈ 750 MHz, *κ*/2*π* ≈ 2.62 MHz and *γ*/2*π* ≈ 3.5 MHz are predictively achievable in ref. 67. For such parameters, the fidelity of our scheme is larger than 99.0%, thus, the present shortcut scheme is robust against both cavity decay and atomic spontaneous emission.

## Additional Information

**How to cite this article**: He, S. *et al.* Efficient shortcuts to adiabatic passage for three-dimensional entanglement generation via transitionless quantum driving. *Sci. Rep.* **6**, 30929; doi: 10.1038/srep30929 (2016).
